# Is a tourniquet necessary in arthroscopic anterior cruciate ligament reconstruction? A randomized controlled study protocol

**DOI:** 10.1097/MD.0000000000023724

**Published:** 2021-02-05

**Authors:** Weifeng Liao, Xinning He, Zhiyong Du, Yi Long

**Affiliations:** a Department of Spine Surgery, the Orthopedics Hospital of Traditional Chinese Medicine Zhuzhou City; b Department of Joint Surgery, the Affiliated Zhuzhou Hospital Xiangya Medical College CSU, Zhuzhou, Hunan, China.

**Keywords:** anterior cruciate ligament reconstruction, tourniquet, randomized controlled trial, study protocol

## Abstract

**Background:**

In the past few decades, the number of surgery of anterior cruciate ligament reconstruction (ACLR) implemented in the outpatient centers has dramatically enhanced. There is still a lack of effective randomized controlled trials in the literature to demonstrate the effectiveness of tourniquets. As a kind of prospective clinical trial, this research protocol is conducted to compare the results of ACLR with and without the use of a tourniquet.

**Methods:**

All the patients aged 18 or over who underwent the selective primary anterior cruciate ligament reconstruction in our hospital from November 2020 to January 2022 are eligible to take part in our experiment. Exclusion criteria are history of peripheral neuropathy, pregnancy, lumbar radiculopathy, or surgery to the injured or contralateral knee. After the written informed consent is given, the patients participating in the study are randomly assigned to the tourniquet group (group 1) and the tourniquet free group (group 2) on the day of operation, through utilizing the computer-generated random table with 10 members in each group. And the assignments were kept in an opaque and sealed envelope. Any comments on visual difficulties in the process of operative time, arthroscopy, complications, and total bleeding from suction and drainage, as well as the reduction of postoperative hemoglobin are assessed as the parameters. The software of SPSS v. 24 is applied for all the statistical analyses.

**Results:**

This protocol will provide a reliable theoretical basis for the following research.

**Trial registration:**

This study protocol was registered in Research Registry (researchregistry6240).

## 1 Introduction

In the past few decades, the number of surgery of anterior cruciate ligament reconstruction (ACLR) implemented in the outpatient centers has dramatically enhanced. For instance, about 43 percent of ACLR were conducted in 1994 in the outpatient clinics, which rose to more than 94 percent in 2014.^[1,2,3,4,5]^ However, it is not clear whether a tourniquet should be used in ACLR surgery. The advantages of using tourniquets include reducing blood loss in the joints, improving visibility, and thus possibly shortening the operation time.^[6,7,8,9]^ However, there are reports that the use of tourniquets have adverse effects. Disadvantages involve muscle atrophy and weakness, neuropathy, and the increased pain after operation. Some uncommon complications such as the high incidence rate of thromboembolic events and rhabdomyolysis have also been reported.^[10,11,12,13,14,15]^

A number of researches have been implemented to explore the efficiency of tourniquet on the prognosis after receiving ACLR.^[16,17,18,19,20]^ Daniel et al confirmed that tourniquet applications at 12 weeks after operation can delay the recovery of quadriceps femoris strength and lead to more muscle atrophy, but there is no apparent difference at 52 weeks.^[16]^ In the literature, a number of randomized studies on our subject are reported.^[10,21]^ Nicholas et al concluded that using the tourniquets led to a significant decrease in thigh circumference at 3 weeks, but there is no difference in the muscle strength at 6 months.^[10]^ Nakayama et al suggested that there is no difference in function between groups 3 months after operation.^[21]^

There is still a lack of effective randomized controlled trials in the literature to demonstrate the effectiveness of tourniquets. As a kind of prospective clinical trial, this research protocol is conducted to compare the results of ACLR with and without the use of a tourniquet.

## 2 Materials and methods

### 2.1 Patients and design

This trial protocol was approved through the institutional review committee of the Affiliated Zhuzhou Hospital Xiangya Medical College CSU (protocol number 182-15) and it had been prospectively registered with the Research Registry (researchregistry6240). This current report is implemented in accordance with the guidelines of Consolidated Standards of Reporting Trials (CONSORT). All the patients aged 18 or over who underwent the selective primary anterior cruciate ligament reconstruction in our hospital from November 2020 to January 2022 are eligible to take part in our experiment. Exclusion criteria are history of peripheral neuropathy, pregnancy, lumbar radiculopathy, or surgery to the injured or contralateral knee. After the written informed consent is given, the patients participating in the study are randomly assigned to the tourniquet group (group 1) and the tourniquet free group (group 2) on the day of operation, through utilizing the computer-generated random table with 10 members in each group. And the assignments were kept in an opaque and sealed envelope.

### 2.2 Surgical techniques

Surgery is performed under general anesthesia. All the surgeries are implemented through an experienced author. In the group 1, the tourniquet is inflated to 300 mm mercury and the tourniquet time is recorded. In the group 2, the tourniquet is not inflated. Anatomically single-bundle ACL reconstruction is performed using autogenous bone tendon-bone grafts collected from the ipsilateral knee of patient. The blunt resection of central 1/3 patellar tendon is carried out with a 1/4 inch curved osteotome and a swinging bone saw. Femoral tunnel is independent of tibial tunnel and drilled in the original ACL footprint center through accessory anteromedial portal. Through remaining tibial footprint, the anterior-medial tibial tunnel can be drilled with the posterior reamer. The graft enters the joint through taking the suture ring from femoral tunnel and then passing the sutures from the anterior cruciate ligament graft through suture ring and passing out through lateral thigh. The graft femoral side is fixed with the interference screw; while tibial side is fixed with suspensory fixation or aperture fixation. Before fixing the tibial, graft is tensioned in the range of 10 to 20 N, and knee is circulated fifteen times, if necessary, the graft is kept tensioned. After tensioning the graft, an arthroscope is inserted immediately at the joint to ensure that there is no roof and wall impingement.

### 2.3 Rehabilitation

We utilized an identical program of rehabilitation for the 2 techniques, emphasizing the restoration of quadriceps function and complete extension as quickly as possible. The program of accelerated rehabilitation is started immediately after finishing the operation. And the full weight-bearing is encouraged where permitted. Utilizing the compression and cooling systems from day 1 until the swelling is evidently reduced. In the first 6 weeks, a brace is applied to allow full flexion. And the quadriceps strengthening is limited to the first 3 months of closed exercise of kinetic chain. The water jogging, cycling and proprioceptive training are started after 3 weeks and lasted for 3 months. Jogging is allowed at the earliest 3 months after operation. It is recommended to resume the unrestricted physical activity for at least 6 months, involving some pivotal sports such as skiing or football.

### 2.4 Outcomes

Any comments on visual difficulties in the process of operative time, arthroscopy, complications, and total bleeding from suction and drainage, as well as the reduction of postoperative hemoglobin are assessed as the parameters. The patients evaluated pain after operation (i.e., visual analogue scale) is scored the next day according to grading scale. The visual analogue scale is horizontal line of 100 millimeters in length, with both ends fixed by the word descriptors. The patients mark the point on the line that they thought best expresses their perception of their present state. The score of visual analogue score is measuring via determining the millimeter from left end to the patient's marker point.

### 2.5 Statistical analysis

The software of SPSS v. 24 is applied for all the statistical analyses (IBM Corp., Armonk, NY, USA). The descriptive statistics of clinical characteristics and demographic characteristics are expressed in terms of the mean standard deviation of the continuous scale variables. Student *t* test is applied for the difference between the continuous scale variables of normal distribution, and Wilcoxon rank sum test is utilized for the difference between the non-normal variables. The Fisher exact test and Pearson Chi-Squared test is used to determine the correlation between the categorical variables. On the basis of intention-to-treat principle, all the analyses can be carried out.

## 3 Discussion

There is still a lack of effective randomized controlled trials in the literature to demonstrate the effectiveness of tourniquets. As a kind of prospective clinical trial, this research protocol is conducted to compare the results of ACLR without or with using the tourniquet. It is speculated that the use of tourniquet can shorten the operation time via improving the visual effect, whereas the postoperative bleeding and increased complaints, resulting in postoperative early recovery disorders. This protocol will provide a reliable theoretical basis for the following research.

## Author contributions

**Conceptualization**: Yi Long.

**Data curation**: Weifeng Liao, Xinning He, Zhiyong Du.

**Formal analysis**: Weifeng Liao, Xinning He.

**Funding acquisition**: Yi Long.

**Investigation**: Weifeng Liao, Xinning He.

**Methodology**: Zhiyong Du.

**Resources:** Yi Long.

**Software**: Zhiyong Du.

**Supervision**: Yi Long.

**Validation**: Zhiyong Du.

**Visualization**: Xinning He.

**Writing – original draft**: Weifeng Liao.

**Writing – review & editing**: Yi Long.
